# Parenting Programs to Reduce Recurrence of Child Maltreatment in the Family Environment: A Systematic Review

**DOI:** 10.3390/ijerph192013283

**Published:** 2022-10-14

**Authors:** Luisa Morello, Marcella Caputi, Simona Scaini, Barbara Forresi

**Affiliations:** 1 Child and Youth Lab, Sigmund Freud University of Milan, Ripa di Porta Ticinese 77, 20143 Milano, Italy; 2 Department of Life Sciences, University of Trieste, Via Weiss 21, 34128 Trieste, Italy

**Keywords:** child physical abuse, child maltreatment, parenting programs, tertiary prevention

## Abstract

Physical maltreatment is a public health issue affecting millions of children in their lifetime, with a high risk of recurrency. Although there are several parenting programs (PPs) available, existing reviews on their effectiveness in preventing physical abuse recurrences have many limitations. The current systematic review aims at (1) providing a summary of evidence on the effectiveness of behavioral/cognitive–behavioral PPs in preventing physical re-abuse; (2) extending previous reviews by including reduction of child maltreatment recurrence as the main outcome but also focusing on the effect of PPs on maltreatment risk, parent and child psychopathology, and parent–child relationship; and (3) including only RCT with at least one follow-up. A PRISMA-compliant systematic review was performed in the EBSCOhost and PUBMED databases. In total, 93 articles were identified, of which 8 were included in the review. Among them, three reported a significant reduction in recidivism rates and maltreatment risk, and five improvements in parent–child relationships. Although limitations arise from methodological heterogeneity across studies, there is some evidence that some brief and manualized cognitive behavioral PPs can reduce the recurrence of child physical maltreatment and improve parent–child relationships. More studies are needed to give further support to PP effectiveness in protecting children from recurrent maltreatment.

## 1. Introduction

Child physical maltreatment can be defined as the intentional use of physical force, involving hitting, shaking, throwing, burning, suffocating or other ways to cause physical harm by a parent or caregiver against a child [1,2]. According to WHO, child physical maltreatment is a public health issue that concerns one out of four children in their lifetime [3]; studies based on self-reported data suggest similar estimates, indicating a rate of 22.6% for physical abuse, with a high risk of recurrency [4]. However, child maltreatment is an underrated phenomenon because of different reasons: among the others, it occurs privately, it is not self-reported by parents, and even when detected, it is often not reported to public authorities [5].

Data showed short and long-term consequences of physical maltreatment on children’s health. In particular, physical maltreatment is associated with externalizing symptoms, aggressive behaviors, delinquency, and drug abuse [5,6,7]. Moreover, maltreated children are at increased risk of developing PTSD, depression, and anxiety and of committing suicide [5,8,9]. Furthermore, associations between maltreatment and cognitive delays and poor school achievement have been recognized [5,7], as well as long-term physical health effects (such as obesity) throughout adolescence and adulthood [5,6]. Neurobiological alterations have been also observed in maltreated children, mainly concerning the neuroendocrine system [10,11]. Physical abuse recurrences often foster a downward spiraling trajectory for children and families, with increased risk of severe injury, and considerable direct and indirect costs, given its impact on health services, child welfare, and criminal justice [5]. Therefore, understanding how to reduce its recurrence is of paramount importance.

In the last two decades, several parenting programs (PPs) have been developed for intervening with families and improving parent–child relationship. Theoretical underpinnings of PPs stem from different conceptualizations of child maltreatment [12].

Among behavioral theories, Patterson’s coercion theory is one of the most accredited, according to which child physical maltreatment results from a pattern of repetitive coercive and violent parent–child interactions that escalate exponentially [13,14]. With his Social Learning Theory, Bandura extended the behavioral approach by introducing the role of vicarious learning or “modeling” [15,16]. According to this theory, abusive and aggressive behavior can be learned by observing abusive parenting, without direct reinforcement [17]. Cognitive models, such as the Social Information Processing Theory, draw attention to the role of cognitive processes that interfere with a healthy parent–child relationship, including parental executive functioning deficits, rigid schemas, unrealistic expectations, and misleading beliefs about themselves and the child [18,19].

Other theories applicable to child maltreatment include Bowlby’s theory focused on attachment organizations [20]; theories on parental stress, suggesting interactions between stress and poor parental coping skills [21,22]; and theoretical models of parental emotion regulation, showing associations between parents’ increased sensitivity to child negative affect, angry emotional responses, and abusive parenting [23,24]. Other conceptualizations highlight the key role of parental depression/substance abuse [17,25], parents’ young age, low education level, and poverty [17,25,26]. Finally, the ecological model based on Bronfenbrenner’s theory (1979) [27] suggests the crucial role played by social and environmental factors in influencing parent–child relationship [28,29].

To date, research mainly focused on assessing parenting programs effectiveness as a secondary prevention intervention, thus considering at-risk families where maltreatment has not occurred yet [30,31,32,33]. Less is known about the impact of parenting programs as a tertiary prevention intervention, on families with prior reports of child physical maltreatment, aimed at reducing the recurrence of physical abuse.

Existing reviews on tertiary prevention interventions provided promising results, highlighting the potential effectiveness of behavioral or cognitive behavioral PPs [2,34,35,36]: these programs’ main goal is breaking the coercive cycle that characterizes violent parent–child relationships, by increasing positive parenting skills, parental executive functioning, cognitive flexibility, and parental sensitivity [12,36]. However, although encouraging, this evidence shows important limits: some of these reviews are dated, others have a too wide scope, or a high level of heterogeneity is included in the studies. Barlow et al., published a systematic review in 2006 suggesting that PPs can be an effective method to prevent new incidents of child physical abuse [37]; however, their review was focused on both maltreatment and neglect. Similarly, in their 2015 systematic review and meta-analysis, Chen and Chan highlighted the effectiveness of PPs in reducing recidivism of child maltreatment [38], but their work covered all types of child maltreatment. The systematic review by Santini and Williams (2016) limited the investigation to the effectiveness of PPs in reducing parental use of corporal punishment [39]. Vlahovicova and colleagues, in their 2017 systematic review and meta-analysis, showed modest but significant effectiveness of PPs in reducing child physical abuse recidivism [36]. This review was focused on families with a referred history of child physical abuse, considered parenting programs based on the Social Learning Theory and included only randomized controlled trials. Secondary outcomes of PP interventions, however, were not deeply investigated.

The current systematic review responds to the general objective of making a step forward in the understanding of *what works for whom* [40]. More specifically, it aims at the following:

1. Providing a summary of evidence on the effectiveness of behavioral/cognitive–behavioral PPs in preventing physical re-abuse in children (0–18);
2. Extending previous reviews by including a reduction in recurrence as the main outcome measure but also focusing on the effect of PPs on secondary outcomes such as a reduction in maltreatment risk, a reduction in parental and child psychopathology, and an improvement in parent–child relationships;
3. Overcoming prior reviews’ methodological limitations by including only randomized controlled trials (with at least one follow-up). Moreover, the present work provides a deep and focused look into the intervention programs, to point out what variables and treatment components support their effectiveness in reducing maltreatment recidivism.

## 2. Materials and Methods

### 2.1. Search Strategy

Preferred Reporting Items for Systematic Reviews and Meta-Analyses (PRISMA) guidelines were used to conduct the present review [41]. To identify the included articles, two databases were searched: Pubmed and EBSCOhost (PsycINFO, PsycARTICLES, PSYINDEX, MEDLINEERIC). The last search was conducted on March 2022. The search string used in the databases search was: (parenting OR family) AND (intervention OR treatment OR therapy OR program OR training) AND (“physical maltreatment” OR “physical abuse”) AND (child OR minor) AND (“randomized controlled trial” OR “randomised control trial” OR RCT). Additional material was identified through exploratory and citation searching. The research was limited to studies written in English, without applying temporal restrictions. Eligibility criteria were established according to PICOS (population, intervention, comparison, outcome measure, and study design) domains. To be included in the present systematic review, articles had to meet the following inclusion criteria:

1. Population: parents or other primary caregivers of children aged 0–18 years. Parents were eligible if they were referred for suspected or substantiated child physical maltreatment, supported by either an official report (police, child protection services or other official agency referral), parents’ or children’s self-reports, or an assessment through standardized instruments. Both suspected and substantiated cases were considered eligible, having the same recidivism risk [42].
2. Intervention: behavioral or cognitive–behavioral parenting programs aimed at reducing the recurrence of child physical maltreatment within families (tertiary prevention).
3. Comparison: the control group could be either an alternative intervention group, treatment as usual, waitlist, or no treatment.
4. Outcome measures: a reduction in physical maltreatment recurrence according to official reports, according to parents’ or children’s self-reports, through standardized instruments, or through maltreatment-related indicators (such as physical punishment or harsh parenting). The following secondary outcomes were included in the present review: reduction in maltreatment risk, reduction in parental and child psychopathology, and improvement in parent–child relationship.
5. Study design: randomized controlled trials with at least one follow-up.

Studies were excluded from the present systematic review when:

1. The sample did not include parents but other subjects (e.g., teachers).
2. The focus was on other forms of child abuse, such as sexual abuse, emotional abuse, and neglect that need specific interventions, as highlighted by NICE guidelines [2,43].
3. The parenting program was not behavioral or cognitive–behavioral in the approach or was used as a primary or secondary prevention intervention.
4. Studies were not focused on the reduction in child physical maltreatment within families as the main indicator of a parenting program’s effectiveness.

### 2.2. Selection Process and Data Extraction

All the records retrieved from the database search were screened by the first author of the present systematic review, according to the eligibility criteria. Firstly, titles and abstracts were screened. Then, full texts of the remaining articles as well as of records retrieved from other sources were read to decide whether to include them.

The following data were extracted from each included study: article identifiers (authors, year of publication, and country where the study was conducted); sample size, gender and median age of parents, and age range of children; duration of follow-up; name, dose, and setting of the intervention group; type of control group; child physical recurrence measure; and re-abuse reports. Moreover, data concerning secondary outcome variables and assessment instruments were extracted and categorized.

## 3. Results

### 3.1. Study Selection

The search strategy applied to the databases yielded 93 articles. Following duplicate removal, a total of 79 records were screened for relevance. Title and abstract screening led to the exclusion of 51 articles. Thus, 28 full-text reports were retrieved and assessed for eligibility. Two studies were excluded because they were focused on practitioners; nine were excluded because they did not consider a tertiary prevention intervention, or the parental program was not behavioral or cognitive–behavioral. Five studies were excluded because they did not focus on the reduction in recurrence of child physical maltreatment or on the assessment of the effectiveness of a parenting program addressing child maltreatment. Finally, four articles were excluded because they were not randomized controlled trials with at least one follow-up.

**Figure 1:**
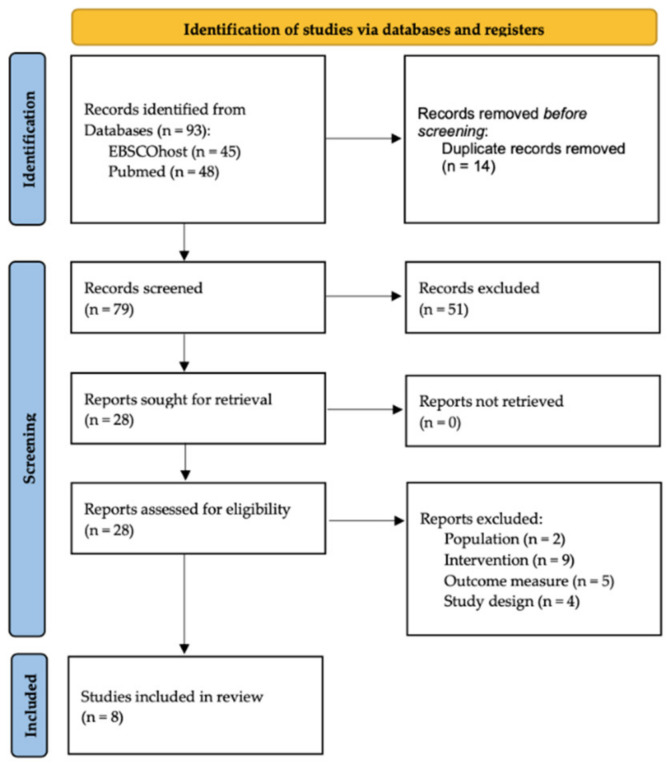
Preferred Reporting Items for Systematic Reviews and Meta-Analyses (PRISMA) flow chart.

In total, eight studies were included in the present systematic review. Figure 1 shows details of the study-selection process.

### 3.2. Characteristics of the Included Studies

Table 1 reports a summary of the characteristics of included studies. Six studies were conducted in the USA, one was conducted in Canada [44], and one was conducted in Brazil [45].

#### 3.2.1. Population

According to the aim and the inclusion criteria of the present review, study samples were parents referred for suspected or substantiated child physical maltreatment. In total, 729 parents and 745 children were involved. The study with the smallest sample involved 35 families, of whom 17 were assigned to the intervention group and 18 to the control one [47]. On the other side, the trial with the largest sample size involved 195 families, of whom 122 were assigned to the intervention group and 73 to the control one [49]. In the case of multiple children, 7 out of 8 studies limited the sample to one child per parent [51]. Among the six studies in which gender was specified, parents were mainly females (ranging from 65% to 100% of the sample) [44,45,46,47,50,51], with three studies involving only mothers [45,47,50]. Parents’ median age was reported in six studies and ranged from 26.65 [50] to 33.02 years old [51].

Age range of children differed consistently among the studies. The trial involving the youngest children considered an age range of 0–7 years old [50], whereas the study with the oldest ones covered an age range of 5–15 years old [49]. Notably, the study with the widest children age range involved children from 0 to 13 years [44].

#### 3.2.2. Interventions

In accordance with the eligibility criteria, all the trials used behavioral or cognitive behavioral parenting programs as tertiary prevention interventions. While programs varied in components and delivery settings, they were all focused on teaching, practicing, and promoting parenting skills; child’s behavioral and emotional management strategies; effective communication skills; and positive parent–child interactions. In two studies, PPs also aimed at facilitating the connection between families and other community resources, based on an ecological model [44,50].

Three studies used a CBT manualized intervention protocol: individual child and parent CBT [48], Combined Parent–Child CBT (CPC-CBT) [51], and Alternatives for Families: A Cognitive Behavioral Therapy (AF-CBT) [49], combining CBT with Family Therapy techniques. Another two studies used manualized parenting programs: Jouriles and colleagues used the cognitive–behavioral Project Support intervention [47], while Chaffin and colleagues used two behavioral-based interventions, the Parent–Child Interaction Therapy (PCIT), and Enhanced Parent–Child Interaction Therapy (EPCIT) (providing individualized enhanced services in addition to the PCIT program) [46].

In two studies, PPs were developed according to evidence-based home visitation models: Healthy Families New York (HFNY) [50] and Home Visitation by a nurse [44]. The last study used an ad hoc PP combining positive parenting techniques, live coaching, and video feedback [45].

In four trials, the PP required the joint presence of the parent and child [44,46,47,50]. One study involved parents and children separately [48], and two others involved them in both individual and joint sessions [49,51]; one study included parents only [45].

The duration of the programs varied considerably, ranging from two months and a half [45] to five years [50]. PPs were mainly delivered through weekly sessions lasting from 50 min to 1.5 h that took place at home [44,47,50], in clinics [45,46,49,51] or both [48].

#### 3.2.3. Comparison Groups

Four trials used treatment-as-usual control groups [44,47,49,50]. In Jouriles et al., one part of the control group received no treatment, while the other received an alternative parenting program and, in some cases, individual therapy sessions [47]. In Kolko et al., the control group received a routine treatment administered by the referring service [49]. In Lee et al., the control families received information and referral to other community services [50]. Standard services in MacMillan et al., offered an assessment of recidivism risk, parenting education, and referrals to other parent education programs [44].

Three trials provided the control groups with alternative treatments [46,48,51], such as the Standard Community Group, a group-based psychoeducation program [46], a Family Therapy [48], and a Parent-only Cognitive Behavioral Therapy [51]. Only one trial used a wait-list control group [45].

#### 3.2.4. Follow-Up

The length of follow-up varied greatly among the trials, ranging from 3 months [51] to 2.3 years [46] after treatment. A couple of studies planned multiple follow-up: one study at 6 and 12 months [49], while another one at 4 and 8 months [47]. In the remaining studies, follow-ups were at 4 months [45], 1 year [44,48], and 2 years [50]. Re-abuse reports were detected at follow-up in six studies [44,46,47,49,50,51], while two studies measured recurrence only at post-treatment [45,48].

### 3.3. Primary Outcome Measure: Re-Abuse Reports

Most of the trials (six) assessed the recurrence of child physical maltreatment through official reports from Child Protective Services (CPS) [47,50], Child Welfare System (CWS) [46,49], or Child Protection Agencies (CPA) [44,51]. In Kolko [48], maltreatment recurrence was measured through children’s and parents’ self-report, whereas in Santini et al. [45], it was measured through observations of negative parent–child interactions.

Overall, trends of reductions in child physical maltreatment reports were observed in all the trials according to within or between comparisons. In total, three studies reported statistically significant reductions in child physical maltreatment. One study reported a significant reduction in the intervention condition at follow-up [49], another one reported a significantly greater reduction in the intervention group than in the control one at follow-up [46], and one study reported both within- and between-group differences at post-test, showing improvements in the intervention conditions [48].

#### 3.3.1. Within-Group Differences

Three studies observed within-groups differences in child physical maltreatment re-reports [48,49,51]. While Runyon et al. [51] observed new cases of physical maltreatment at follow-up, the others reported significant reductions in the intervention condition [48,49].

Kolko compared pre-treatment to the early (first four sessions) and late (last four sessions) phases of treatment: parents reported significant reductions in the intervention condition (from 45.5% to 9.1%, *p* < 0.03) [48]. In the study by Kolko et al., trends of reductions could be observed in the control condition, but only in the intervention group, they were significant (from 17% at baseline to 5.3% at follow-up, *p* = 0.01) [49].

#### 3.3.2. Between-Group Differences

Six studies observed between-group differences in child physical maltreatment re-reports [44,45,46,47,48,50], two of which [46,48] reported statistically significant changes. In the last four session of the treatment, Kolko observed a statistically significant difference in physical maltreatment reports between the intervention and the control group: both children (*p* < 0.007) and parents (*p* < 0.04) reported a lower number of cases in the intervention group [48]. Chaffin et al., observed a significant difference between the first intervention condition and the control group at follow-up (19% of new reports in the first intervention condition vs. 49% of new reports in the controls) (*p* < 0.02) [46].

Even in the four studies that did not reach a statistical significance, lower rates of child maltreatment were also observed in the intervention group at follow-up [44,45,47,50].

Interestingly, after the parenting program, two studies observed higher rates of hospital reports [44] or higher rates of mothers reported for child maltreatment [50]. In explaining these findings, the authors introduced the “surveillance bias”, which refers to increased outcome-related investigation [52].

### 3.4. Secondary Outcomes (Proxies for Child Maltreatment)

Table 2 reports a summary of assessment measures of secondary outcomes used in each study included in the present systematic review.

#### 3.4.1. Maltreatment Risk

Among the four studies that analyzed maltreatment risk [44,46,48,49], three observed that parenting programs were effective: one reported a significant pre-post reduction in both conditions [46], one reported a significantly greater reduction in the experimental condition than in the control one at post-test [49], and the last one reported a significant improvement only in the intervention condition, according to both within- and between-group analyses [48].

Kolko et al., observed that, at post-test, parents in the experimental condition showed statistically significant greater reductions in abuse risk than in the control condition (*p* = 0.03) [49]. It was also observed that parents receiving the intervention program showed greater reductions in threats of force at follow-up, although the difference was not statistically significant.

Reductions in parental anger were found by Kolko, who observed a significantly greater reduction in the intervention group compared to the control one (*p* < 0.05), according to parents’ reports [48]. Regarding family problems, statistically significant greater reductions were observed in IG (than CG) according to children’s reports but not according to parents’ reports (*p* < 0.05).

While Macmillan et al., observed some improvements in maltreatment risk from baseline to follow-up in both groups [44], Chaffin et al., [46] did not find post-test significant differences in maltreatment risk.

#### 3.4.2. Parent–Child Interactions, Parenting, and Family Functioning

Five out of eight studies reported significant improvements of the parent–child relationship in the intervention groups, according to within- or between-group comparisons [45,46,47,49,51]. Three studies detected significant pre-post improvements in the intervention conditions [45,47,51]: Santini et al., reported significant increases in positive interactions (*p* = 0.002) [45]; Jouriles et al., observed reductions in reports of harsh parenting (*p* = 0.01) [47] as well as in the perceived inability to manage childrearing responsibilities (*p* = 0.001) at the follow up. Runyon et al., detected significant pre-post improvements in corporal punishment in both groups (*p* < 0.01), according to parents’ and children’s reports [51] and significant pre-post improvements in positive parenting in the intervention group, that remained at follow-up.

No statistically significant pre-post differences were found by three studies [44,46,49], although one of them observed improvements in family functioning in both groups [44].

According to between-groups analyses, greater improvements in parents involved in the intervention program were detected in parent–child interactions [45,46,47,49], in parenting [47,51], and family functioning [49].

Chaffin et al., observed a reduction in parents’ negative behavior (*p* < 0.01) [46], Santini et al., observed more positive interactions (*p* = 0.011) [45], while Jouriles et al., observed reductions in ineffective parenting (*p* < 0.05) and harsh parenting (*p* < 0.05) [47]. Kolko et al., found that, following the parenting program, children reported lower rates of minor physical assault directed to caregivers at post-test (*p* = 0.01) and follow-up (*p* = 0.001) [49]. Runyon et al., observed increases in positive parenting in the intervention group (*p* < 0.01) and a decrease in corporal punishment in the control group (*p* < 0.05, although reductions were observed in both conditions) [51]. Changes remained at follow-up.

In accordance with children’s reports, although significant reductions in corporal punishment were observed in both conditions, no between-group differences were detected. Jouriles et al., observed significant reductions in the perceived inability to manage childrearing responsibilities in the intervention condition from pre-test to follow-up (*p* < 0.05) [47]. Kolko et al., and MacMillan et al., did not find any significant differences between the two conditions (although Macmillan and colleagues observed improvements in both groups) [44,49]. However, Kolko et al., reported that IG showed greater reductions at post-test (but not at follow-up) in general family dysfunction (*p* = 0.03) and family conflict (*p* = 0.03) [49].

#### 3.4.3. Parental Psychopathology

All three studies investigating parental psychopathology reported significant pre-post changes [45,46] and from pre-test to follow-up [45,47]. Three studies also observed between-group differences [45,46,47].

While Chaffin et al., found pre-post reductions in distress and loneliness within all groups (*p* < 0.05) [46], Santini et al., reported reductions in depression within the intervention condition (*p* = 0.014), that were also significant at follow-up (*p* = 0.002) [45]; Jouriles et al., found reductions in psychological distress within the intervention group at follow-up (*p* < 0.05) [51].

Chaffin et al., found higher pre-post reductions in depression in the EPCIT group (IG2) than in the other two conditions (*p* < 0.05) [46]. Jouriles et al., observed pre-post decreases in psychological distress in the intervention group, but no statistically significant differences between groups were detected [47]. Santini et al., detected higher depressive scores at follow-up in the control condition (*p* = 0.014) [45].

#### 3.4.4. Child Functioning

Among the five studies analyzing child functioning [44,45,46,49,51], four reported statistically significant changes according to within or between comparisons [45,46,49,51]. Two studies reported significant pre-post changes [46,49], and two were also reported at follow-up [45,51].

Chaffin et al., found a significant decrease in parents’ reports of children internalizing and externalizing scores in all groups [46]. Runyon et al., reported a reduction in PTSD symptoms (*p* < 0.001) and in parents’ reports of children’s internalizing problems (*p* < 0.01) in both conditions [51].

Santini et al., saw that parents in the intervention group reported lower levels of children’s conduct problems at post-test (*p* = 0.024), that were maintained at follow-up (*p* = 0.000) [45]. Kolko et al., detected a significant decline in PTSD in the intervention group at post-test (*p* = 0.007) but not at follow-up [49]. Macmillan et al., observed improvements over time in child functioning in both groups, without reporting further analyses [44].

Five studies observed between-groups differences in child functioning [44,45,46,49,59], three of which statistically significant: changes were observed at follow-up [49], and both at post-test and follow-up [45,51]. Runyon et al., detected significant differences in PTSD symptoms, with lower symptoms in the intervention group at post-test (*p* < 0.01), confirmed at the 3 months follow-up [51]. Santini et al., observed that children’s total difficulties and conduct problems were lower in the intervention group at follow-up (*p* = 0.000) [45]. Kolko et al., found a greater reduction in child problems in the intervention group over the follow-up period (*p* = 0.01) [49]. The remaining studies did not find any significant difference in child functioning [44,46].

## 4. Discussion

The present systematic review aimed at providing a summary of evidence on the effectiveness of behavioral and cognitive behavioral parenting programs (PPs) in preventing child physical re-abuse. Only randomized controlled trials, with at least one follow-up were included. While recidivism was the primary outcome, this work also focused on PP secondary outcomes: maltreatment risk, parent–child relationship, parental psychopathology, and child functioning.

The results suggest that parenting programs are promising interventions within physically abusive families. In fact, reductions in reoccurrence of child maltreatment were observed in all the trials, although reaching statistical significance in three out of eight studies [46,48,49].

Parenting programs resulting in a significant reduction in physical maltreatment recidivism shared common characteristics: they were specifically focused on abusive parents and involved children throughout the intervention; targeted at families with children aged between 4 and 15 years; and brief, with their duration ranging from 4 to 6 months. All these interventions were based on manualized protocols: one of them based on a behavioral [46] and the other two on a cognitive–behavioral approach [48,49].

A deeper analysis of secondary outcomes, strictly related to child maltreatment, may help understand how PP can be beneficial on multiple levels. Among the four studies that analyzed maltreatment risk, three reported significant reductions in the intervention condition at post-test [46,48,49], suggesting a potential decrease in re-abuse risk. As maltreatment risk is predictive of children’s long-term cognitive and socio-emotional adjustment [76], it is important to include this variable among the indicators of parenting program’s effectiveness.

Five out of eight studies reported significant improvements of the parent–child relationship in the intervention group [45,46,47,49,51], suggesting that PP are also useful to increase positive parent–child interactions [45] and positive parenting [51] and to reduce harsh parenting [47], parents’ negative behavior [46], ineffective parenting and perceived inability to manage children [47], physical assault to caregivers, family dysfunction and conflict [49].

In the studies that found a significant reduction in recidivism rates, a decrease in maltreatment risk was also observed [46,48,49]; moreover, two reported an improvement in parent–child relationships [46,49]. These findings seem to confirm that a change of parent–child relationship patterns [77] is crucially implicated in the recidivism reduction of child physical abuse, representing a key aspect in the treatment of maltreating families [19]. They are also consistent with studies showing that a reduction in coercive parent–child interactions mediates physical abuse recidivism [14,19,46].

Three out of four studies that measured parental psychopathology (mostly depression) reported significant improvements in the intervention conditions [45,46,47]. Consistently, Kolko showed a positive correlation between parental depression and the use of physical discipline/force [48], supporting the theory that recognizes parental psychopathology, in particular maternal depression, as a risk factor for child maltreatment [78]. However, treating parental psychopathology within a PP may be a confounding factor for parents involved in a program in which the main aim is the reduction in parent–child coercive interactions [46]. Parental psychopathology may be a critical aspect [47], but it might be treated outside the parenting program.

All five studies measuring child functioning reported improvements among conditions. In particular, reductions in externalizing symptoms [45,49] and PTSD symptoms [49,51] were observed. These results underline the potential beneficial effect that PP could have on maltreated children that are at increased risk to develop mental health disorders, as reported by previous literature [5]. Runyon and colleagues suggested that it could be useful to involve children in the intervention, especially when they report PTSD symptoms [51]: the Combined Parent–Child CBT they used offers the opportunity to focus on the development of a trauma narrative and involves an abuse clarification process that could help children overcome their PTSD.

More specifically, the present review gives further support to the literature showing that the reduction in recidivism is related to a decrease in parents’ negative behaviors and parent–child conflicts as well as to an increase in positive parenting [19]. Consistently, a correlation between ineffective parenting and parental use of physical punishment [48], as well as a mediating role of parenting behaviors between intervention and maltreatment perpetration were observed by researchers [50].

Another interesting finding is that parenting programs focused on parenting abilities without enhanced services may be sufficient to reduce child maltreatment recurrence. This is in accordance with previous evidence showing that treatments specifically addressing abusive parenting and directly related aspects were more effective than those with wider aims [37]. As highlighted by Chaffin et al., interventions with a broad focus might reduce parents’ interest in the program or may divert parents’ attention to other less relevant objectives [46].

Involving children in the program may be also important not only to enhance their psychological well-being but also to lessening parental use of physical force. This was suggested by correlational analyses reported by Kolko (1996) and by Runyon and colleagues, showing greater improvements in positive parenting when children participated in the treatment [48,51]. Having children engaged throughout the program appears to be beneficial to improve the parent–child relationship and to break the coercive cycle, firstly by promoting more effective communication and by encouraging constructive and supportive interactions [79].

Taken together, the results of the present systematic review indicate that there is some evidence of effectiveness for behavioral/cognitive behavioral PPs in preventing the recurrence of child physical abuse. Their potential beneficial effects are also related to a reduction in maltreatment risk and parental psychopathology as well as to an improvement in parent–child relationships [36].

### 4.1. Clinical Implications

The findings of the present work suggest that more effective PPs are those specifically addressing physically abusive parenting. For this reason, PPs should focus less on parental psychopathology, which could be addressed by other services. Along with a narrow focus on maltreating parenting, the results also suggest that PPs should provide modules involving children as active participants in individual or joint sessions with parents. This structure would allow parents to have a better understanding of children’s behaviors and emotions and to practice positive parenting skills under the supervision of experts, strengthening the learning process and the maintenance of skills over time [36,45,51]. Involving children throughout the treatment would facilitate changes in coercive parent–child interactions, which represent the key point to promote the reduction of recidivism.

Effective interventions turned out to be short and, for this reason, also cost-effective. Some evidence (coming from studies making a comparison between the first and later sessions of the treatment) showed that crucial changes might occur in initial phases of treatment; therefore, too long an intervention may not add any further improvement [19,46,49]. More studies are needed to explore these findings.

It is also recommended to carry out a good assessment of families to be involved in the program in order to identify specific needs and to choose the best treatment. Some families, in fact, may benefit from a behavioral treatment, while others may need a cognitive–behavioral approach, with a focus on thoughts patterns and processes.

Although it is still difficult to identify a clear evidence-based method for preventing the recurrence of maltreatment, the potential value of manualized interventions such as Parent–Child Interaction Therapy and Alternatives for families: A Cognitive Behavioral Therapy is strongly recognized [80,81].

### 4.2. Limitations and Future Directions

The current systematic review has the main strength of summarizing the evidence about interventions that are successful at addressing maltreatment reoccurrence, giving a contribution to the available knowledge about what works for maltreated children.

Study limitation should be recognized and considered: first, the absence of a common definition of child physical maltreatment, not always made explicit by authors, and the use of different parameters to assess maltreatment reduction: some studies used official reports based on a juridical definition, while others were based on clinical definitions.

Second, although the complexity of treating physically abusive families is wide recognized [49], studies are still affected by methodological limitations such as significant dropout rates, low completion rates, small sample sizes, and short follow-up [44,47,49,50]. Several authors recognized that the presence of attendance barriers—e.g., physical (e.g., transportation costs and work schedules) or social (e.g., stigma) barriers, or factors such as high criticism at pretreatment, low engaging provider’s style, and low socioeconomic status [82,83]—could limit adherence to treatment and lead to dropouts [45,51]. Given the importance of a good engagement in the effectiveness of parenting programs, future studies should further explore these issues.

Other limits concern the study samples, the assessment measures, and the follow-up. The present review confirms that the maltreatment literature is characterized by a limited presence of fathers: future studies should address this issue, as their involvement is crucial to obtain a real understanding of PP effectiveness. There was also a lack of information about participant characteristics (such as being married or divorced, living together, or being single-parent household) or other contextual factors that could influence the intervention’ success. Moreover, no conclusions can be drawn about parenting programs’ effectiveness on children of different age groups. Although previous literature showed that newborns and pre-school children are at increased risk for recurring maltreatment, there is a gap in effective treatments available for this age range [84,85].

The heterogeneity in the measures adopted to assess primary and secondary outcomes makes it difficult to effectively compare findings; furthermore, the use of self-report could result in reporting bias and possibly in an underestimation/overestimation of some outcomes. In addition to this, due to short follow-up periods, it is still difficult to understand whether treatment outcomes are sustainable in the long term.

A final concern is that parenting programs included in the present review, although behavioral or cognitive–behavioral, show many differences in terms of contents, duration, and professionals providing the programs [48,49].

All these issues should be better addressed in future research. More longitudinal studies are needed to investigate different developmental trajectories of families involved in child maltreatment cases. Many questions are still open: *Under what conditions* are PPs effective? Which variables could explain negative outcomes and why some parents do not show improvements? Are results maintained over time? Children’s age and family factors (such as critical life phases or changes in the family structure), the timing of implementation of the PP, and the role of cultural factors should be better explored.

Investigating these aspects could help practitioners understand which families are most likely to benefit from PP and what strategies could be implemented.

## 5. Conclusions

The current systematic review takes a step forward in understanding what works with maltreating families and gives a contribution to the existing literature on effective interventions for preventing the recurrence of physical abuse. There is some evidence that brief parenting programs, based on a behavioral or cognitive behavioral approach, involving children, and focused on parent–child interactions, can be effective in reducing maltreatment recurrence and improving parent–child relationships.

The recurrence of child maltreatment often fosters a downward spiraling trajectory for children and families, in which the probability of a recurrence increases with each new incident and the time between episodes decreases. These downward spiraling trajectories may lead to an increased risk of serious child injury. Interventions with empirically based effectiveness are therefore of paramount importance to slow or stop this process. Further research is needed on promising practices to identify effective treatment components and to improve their effectiveness.

## Figures and Tables

**Table 1:** Characteristics of the included studies.

1st Author and Year		Country	Population				Follow-Up (After Treatment)
			N	Parents		Children (Age Range)	
Chaffin et al. 2004 [46]		USA	110 IG1: 42; IG2: 33; CG: 35	65% F; *M_age_* = 32		4–12	2.3 years
Jouriles et al. 2010 [47]		USA	35 IG: 17; CG: 18	100% F; *M_age_* = 28.7		3–8	4, 8 months
Kolko, 1996 [48]		USA	38 dyads IG: 21; CG: 17	n/a		6–13	1 year
Kolko et al. 2018 [49]		USA	195 IG: 122; CG: 73	n/a		5–15	6, 12 months
Lee et al. 2018 [50]		USA	104 IG: 52; CG: 52	100% F; *M_age_* IG = 26.65; *M_age_* CG = 26.96		0–7	2 years
MacMillan et al. 2005 [44]		Canada	163 IG: 89; CG: 74	95% F; *M_age_* = 29.2		0–13	1 year
Runyon et al. 2010 [51]		USA	44 P + 60 C IG: 24 P + 34 C; CG: 20 P + 26 C	86% F; *M_age_* = 33.02		7–13	3 months
Santini et al. 2017 [45]		Brazil	40 IG: 20; CG: 20	100% F; *M_age_* = 32.85		4–14	4 months
**Study**	**Intervention Group (IG)**	**Comparison Group (CG)**	**Dose**	**Setting**	**Recurrence Measure**	**Recurrence Reports**	
						**Within Groups**	**Between Groups**
Chaffin et al. 2004 [46]	IG1: PCIT; IG2: EPCIT	Standard community-based parenting group	IG1: 6 months (12–14 ses); IG2: 6 months (IG1 + individual services)	Clinic	CWS reports	n/a	Follow-up IG (19%) < CG (49%) (*p* < 0.02); IG1 (19%) < IG2 (36%), ns; IG2 = CG
Jouriles et al. 2010 [47]	Project support	TAU	8 months: weekly ses (1–1.5 h)	Home	CPS records	n/a	Follow-up IG (5.9%) < CG (27.7%), ns
Kolko 1996 [48]	Individual child and parent CBT	Family Therapy	4 months: 12 weekly ses (1 h) + 1 home ses every 2 clinic ses	Clinic and Home	Children/parents reports	- children reports: early IG/CG = late IG/CG - parents reports: early IG (45.5%) > late IG (9.1%) (*p* < 0.03); early CG = late CG	Late treatment - children rep: IG < CG (*p* < 0.007) - parents rep: IG < CG (*p* < 0.04)
Kolko et al. 2018 [49]	AF-CBT	TAU	6 months	MHS or CWS	CWS reports	Pre IG (17%) > follow-up IG (5.3%) (*p* = 0.01); pre CG (13%) > follow-up CG (3%), ns	n/a
Lee et al. 2018 [50]	HFNY (Home visitation)	Other services	5 years: from weekly ses, diminishing	Home	CPS records	n/a	Follow-up IG (3.3%) < CG (13.4%), ns
MacMillan et al. 2005 [44]	Home visitation by nurses	Standard services	2 years: 1.5 h weekly ses for 6 months + every 2 weeks for 6 months + monthly for 12 months	Home	CPA reports	n/a	Follow-up IG (33%) < CG (43.1%), ns; hospital records: IG (23.6%) > CG (10.8%), sign
Runyon et al. 2010 [51]	CPC-CBT	Parent-only CBT	4 months: 12 weekly ses (1 h), for 16 weeks	Clinic	CPA and parent/child reports	IG: 2 reports at follow-up Sign: n/a	n/a
Santini et al. 2017 [45]	Second unit of *Projecto Parceria* + coaching + VF	Wait-list parenting program 1 month after IG	2.5 months: 8 weekly ses (50 min), 1 ses live coaching (50 min), 1 ses VF	Institution and house-lab	Observations of negative interactions	ns	n/a

Note. AF-CBT = Alternatives for Families: A Cognitive Behavioral Therapy; C = children; CBT = cognitive behavioral therapy; CG = control group; CPA = child protection agency; CPC-CBT = Combined Parent–Child Cognitive Behavioral Therapy; CPS = child protection service; CWS = child welfare system; (E)PCIT = (Enhanced) Parent–Child Interaction Therapy; F = female; HFNY = Healthy Families New York; h = hour; IG = intervention group; MHS = mental health system; n/a = not available; ns = not significant; P = parents; rep = report; ses = session; sign = significance; TAU = treatment as usual; VF = videofeedback.

**Table 2:** Assessment measures of secondary outcomes.

Study	Maltreatment Risk	Parent–Child Relationship			Parental Psychopathology	Child Functioning
		Parent–Child Interactions	Parenting	Family Functioning		
Chaffin et al. 2004 [46]	Child Abuse Potential Inventory (CAP) [53]	Dyadic Parent–Child Interaction Coding System (DPICS-II) [55]	Rigidity Subscale of the Child Abuse Potential Inventory (CAP) [53]	n/a	- Beck Depression Inventory (BDI) [67] - Distress and Loneliness Subscale of the Child Abuse Potential Inventory (CAP) [53]	Behavior Assessment System for Children (BASC) [69]
Jouriles et al. 2010 [47]	n/a	- Videotapes observations - Revised Conflict Tactics Scale (CTS-R) [56]	Parental Control of Child’s Behavior Subscales (PLOC) [61]	n/a	Symptoms Checklist-90-Revised (SCL-90-R) [68]	n/a
Kolko, 1996 [48]	- Child Abuse Potential Inventory (CAP) [53] - Weekly Report of Abuse Indicators (WRAI) [48]	- Conflict Tactics Scale (CTS) [57]	Parenting Scale (PS) [62]	- Cohesion Subscale of the Family Environment Scale (FES) [65] - General Functioning Subscale of the Family Assessment Device (FAD) [66]	Beck Depression Inventory (BDI) [67]	n/a
Kolko et al. 2018 [49]	- Weekly Report of Abuse Indicators (WRAI) [48] - Brief Child Abuse Potential Inventory (B-CAP) [54]	The Parent–Child Conflict Tactics Scale (CTS-PC) [58]	Alabama Parenting Questionnaire (APQ) [63]	- Family Conflict Subscale of the Brief Child Abuse Potential Inventory (B-CAP) [54] - General Functioning Subscale of the Family Assessment Device (FAD) [66]	n/a	- Vanderbilt ADHD Diagnostic Parent Rating Scale (VADPRS) [70] - Child PTSD Symptom Scale (CPSS) [71]
Lee et al. 2018 [50]	n/a	The Parent–Child Conflict Tactics Scale (CTS-PC) [58]	Adult- Adolescent Parenting Inventory (AAPI) [64]	n/a	n/a	n/a
MacMillan et al. 2005 [44]	Child Abuse Potential Inventory (CAP) [53]	Home Observation for Measurement of the Environment (HOME) [59]	Adult-Adolescent Parenting Inventory (AAPI) [65]	General Functioning Subscale of the Family Assessment Device (FAD) [66]	n/a	Revised Behavior Problem Checklist (RBPC) [72]
Runyon et al. 2010 [51]	n/a	n/a	Alabama Parenting Questionnaire (APQ) [66]	n/a	n/a	- PTSD section of the Schedule for Affective Disorders and Schizophrenia for School-Age Children- Present and Lifetime version (K-SADS-PL) [73] - Child Behavior Checklist (CBCL) [74]
Santini et al. 2017 [45]	n/a	Observational Protocol [60]	n/a	n/a	Beck Depression Inventory (BDI) [67]	Strengths and Difficulties Questionnaire SDQ [75]

## Data Availability

Not applicable.
